# Investigation of Thermal–Microstructure–Hardness Relationships in Dissimilar AA5052-H32/AA6061-T6 Friction Stir Welded Joints

**DOI:** 10.3390/ma19071410

**Published:** 2026-04-01

**Authors:** Wenfei Li, Vladislav Yakubov, Michail Karpenko, Anna M. Paradowska

**Affiliations:** 1 School of Civil Engineering, University of Sydney, Sydney, NSW 2008, Australia; weli7086@uni.sydney.edu.au (W.L.); vladislav.yakubov@sydney.edu.au (V.Y.); 2 Australian Nuclear Science and Technology Organization, Lucas Heights, NSW 2234, Australia; 3 Heavy Engineering Research Association, Manukau City Centre, Auckland 2241, New Zealand; mkarpenko@hera.org.nz; 4 School of Aerospace, Mechanical and Mechatronic Engineering, The University of Sydney, Sydney, NSW 2008, Australia

**Keywords:** friction stir welding, dissimilar aluminium, AA6061-T6, AA5052-H32, COMSOL, microstructure, hardness

## Abstract

**Highlights:**

- All simulated peak temperatures were below the melting points of AA5052-H32 and AA6061-T6.
- The COMSOL model predicts thermocouple temperatures with errors within 10%.
- The highest temperatures in the nugget zone, combined with plastic deformation, induced dynamic recrystallisation.
- The minimum grain in the nugget zone was refined about eightfold relative to AA5052-H32.
- The AA5052-H32 TMAZ exhibited the minimum hardness with about 22% reduction.

**Abstract:**

Friction stir welding (FSW) of dissimilar aluminium alloys often results in non-uniform microstructure and hardness distributions due to asymmetric temperature fields and material flow. The objective of this study is to establish a quantitative relationship between thermal history, microstructural evolution, and hardness distribution in dissimilar AA5052-H32/AA6061-T6 FSW joints by combining experimental characterisation with validated thermal modelling. AA5052-H32 and AA6061-T6 plates were welded under five different parameter sets. A thermal finite element model was developed in COMSOL Multiphysics to simulate temperature evolution during welding and was validated using embedded thermocouple measurements, with predicted peak temperatures ranging from 455 °C to 641 °C. Optical microscopy, scanning electron microscopy (SEM), and electron backscatter diffraction (EBSD) were employed to characterise grain structure and dynamic recrystallisation (DRX) behaviour, while Vickers microhardness mapping was used to evaluate the local mechanical response. The results show that DRX occurred in the nugget zone (NZ), leading to significant grain refinement, with a minimum grain diameter of 6.07 µm, representing an approximately eightfold reduction compared with the base material AA5052-H32. In contrast, the thermo-mechanically affected zone (TMAZ) experienced limited recrystallisation due to insufficient plastic deformation and temperature. The lowest hardness was observed in the TMAZ on the AA5052-H32 side, with the hardness reduction of 22% primarily caused by work hardening loss. Hardness was also reduced by 34% on the AA6061-T6 side due to decreased precipitation strengthening caused by high temperatures. This combined experimental–numerical study provides a systematic thermal–microstructure–hardness framework for understanding and predicting local property variations in dissimilar FSW joints.

## 1. Introduction

Aluminium alloys are increasingly pivotal in modern engineering due to their high strength-to-weight ratio and excellent corrosion resistance, making them attractive for lightweight, high-performance structural components. However, joining dissimilar aluminium alloys remains challenging. Conventional fusion welding often leads to porosity, solidification cracking [1,2], high residual stresses, and brittle intermetallic compounds. These issues are particularly pronounced in dissimilar aluminium alloys with different melting points and thermal conductivities [3,4]. Friction stir welding (FSW) is a solid-state joining technique [5] developed by The Welding Institute (TWI) in 1991 [6]. As the material does not melt, FSW offers low heat input, reduced distortion, refined grain structure, and improved mechanical performance [7]. During FSW, a rotating tool generates frictional heat and severe plastic deformation, softening the material and promoting material flow along the joint line while maintaining temperatures below the melting point, thus suppressing typical fusion-weld defects [8]. When the tool rotates and moves forward, the side where the rotation and welding direction are the same is called the “advancing side” (AS). The opposite side, where the rotation direction counters the welding direction, is the “retreating side” (RS). This inherent asymmetry leads to uneven material flow and temperature distribution at the weld, which are particularly pronounced in dissimilar aluminium alloy joints and have a key impact on microstructure evolution and local mechanical properties.

AA5052 is an Al-Mg aluminium alloy that is widely used in the automotive and marine industries due to its excellent weldability and corrosion resistance [9]. AA6061-T6 is a heat-treated aluminium alloy extensively employed in automotive [10], aerospace [11], and railway applications because of its favourable strength-to-weight ratio. When joined by FSW, dissimilar AA5052/AA6061 joints enable the combination of the superior corrosion resistance of AA5052 with the satisfactory structural strength of AA6061, making this material pairing attractive for lightweight engineering structures in automotive, marine, and aerospace.

Existing studies on dissimilar FSW of 5xxx/6xxx aluminium alloys can be broadly categorised in terms of three main aspects: (i) process parameter optimisation and defect formation, (ii) microstructural evolution, and (iii) hardness distribution. Numerous investigations have reported that the balance between tool rotational speed (TRS) and tool traverse speed (TTS) is critical for achieving defect-free joints, particularly due to the strong sensitivity of material flow and heat input in dissimilar systems [12]. Material properties, including thickness and alloy type, also affect heat conduction and weld quality. Verma et al. [13] reported that frictional heat at the tool–workpiece interface governs material softening and flow, making accurate thermal assessment essential for achieving high-strength joints. For dissimilar alloys such as AA5083 and AA6061, an intermediate TRS of around 900 rpm provides the best mechanical performance, whereas lowering the speed to 710 rpm leads to inadequate plastic flow and reduced tensile strength [14]. This demonstrates that dissimilar joints are extremely sensitive to heat input, and even slight deviations in parameters can lead to insufficient flow or defects. Kalemba-Rec et al. [15] further showed that mixing in AA7075-AA5083 joints increases with TRS. However, excessive TRS can cause flash, voids, porosity, or wormholes due to over-softening [16,17]. Lower traverse speeds also increase heat input but lead to defects like tunnelling [18,19]. Doley and Kore [20] reported that, for dissimilar AA5052-H32/AA6061-T6 joints, welding at a TTS of 63 mm/min resulted in higher ductility than at 98 mm/min, which was attributed to enhanced material softening and mixing. Palanivel et al. [21] established an FSW processing window for dissimilar AA5083/AA6351 alloys, demonstrating that defect-free welds are obtained only within moderate heat input ranges, specifically 800–1200 rpm and 45–85 mm/min.

A synthesis of the literature indicates that the nugget zone (NZ), thermo-mechanically affected zone (TMAZ), and heat-affected zone (HAZ) constitute the weld region in FSW [22,23]. In the NZ, dynamic recrystallisation (DRX) forms fine equiaxed grains that enhance hardness and mechanical performance [24]. The TMAZ experiences lower heat input and heterogeneous plastic flow, producing a mixture of distorted laminar grains and partially recrystallised structures. In contrast, the HAZ undergoes grain coarsening without recrystallisation, and its morphology remains similar to the base material (BM) due to limited plastic deformation [7]. Welding parameters strongly influence grain refinement, primarily through their effect on stirring intensity and heat input, which together govern grain size evolution across the weld zones [25]. Clear microstructural asymmetry also exists between the advancing and retreating sides, with the latter typically showing more distinct SZ-TMAZ boundaries [26]. Microhardness variation across FSW regions is governed by grain size and precipitate behaviour. NZ has a higher hardness than TMAZ and HAZ within the weld region due to its finer grain size [27]. Unlike similar FSW joints that often exhibit an approximately W-shaped hardness profile, dissimilar joints do not necessarily show a symmetric W-shape. For example, Baghdadi et al. [28] and Chen et al. [29] both reported that hardness variations in dissimilar 5xxx/6xxx aluminium alloy joints are often asymmetric or fluctuating, reflecting the distinct responses of the constituent alloys.

Previous studies have primarily focused on post-weld experimental characterisation. For example, Farhang et al. [30] systematically investigated the correlation between microstructure, residual stress, and mechanical properties in friction stir welded AA2024-T6 joints. While such approaches offer important insights into joint behaviour, they do not adequately capture how asymmetric thermal histories govern local microstructural and hardness evolution in alloys with distinct strengthening responses. In particular, the combined effects of work-hardening loss in 5xxx alloys and precipitate dissolution or coarsening in 6xxx alloys under non-uniform thermal cycles remain insufficiently investigated. Addressing this gap is the main purpose of this study using a thermal model.

Current COMSOL models of FSW have predominantly focused on similar aluminium alloys. Vignesh et al. [31] numerically investigated the transient thermal behaviour during FSW of AA6061-T6 and reported that the peak temperature is strongly governed by the tool shoulder diameter rather than the pin diameter, while Lemi et al. [32] demonstrated that increasing axial force raises the temperatures on both the top and bottom surfaces of the weld. Abotaleb et al. [33] further employed a coupled thermo-mechanical COMSOL model to predict the temperature distribution and microhardness during FSW of Al-T6. However, existing COMSOL studies focused primarily on thermal fields, without connecting them to experiments to analyse the effect of temperature on the microstructure.

In contrast to previous studies on dissimilar 5xxx/6xxx aluminium alloys, this paper aims to elucidate the microstructure and mechanical properties of dissimilar AA5052-H32/AA6061-T6 joints produced by FSW through validated thermal finite element modelling and detailed experimental characterisation. The model is developed in COMSOL Multiphysics version 6.1 to simulate the welding process and examine the relationship between thermal history, microstructural evolution, and hardness variation across the weld. This study reveals an asymmetric softening mechanism jointly governed by work-hardening loss in AA5052 and precipitate evolution in AA6061, providing a theoretical basis and a predictive framework for optimising the FSW process of dissimilar aluminium alloys.

## 2. Methodology

### 2.1. Experiment

In this study, AA6061-T6 and AA5052-H32 aluminium alloys were selected as the base metals for the FSW joints of dissimilar materials using Stirweld FSW welding head [x] (Stirweld, Rennes, France). Table 1 lists the chemical compositions of the two alloys. AA6061-T6 contains higher silicon, copper and chromium, which is conducive to improving its strength through heat treatment, while AA5052-H32 contains a higher proportion of magnesium, which improves its corrosion resistance and cold working properties.

**Figure 1:**
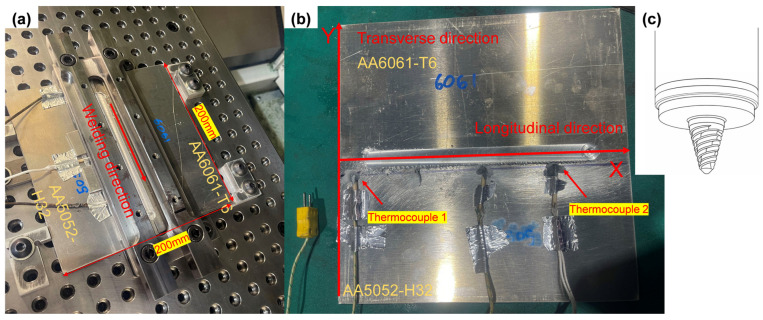
(**a**) Welded sample of AA6061-T6 and AA5052-H32; (**b**) experimental setup showing the thermocouple placement. (**c**) Schematic of the FSW tool.

During welding, AA6061-T6 was positioned on the AS and AA5052-H32 on the RS, as AA6061-T6 is more sensitive to heat and was placed on the higher-strain AS to assess softening associated with precipitation, while AA5052-H32 was positioned on the lower-strain RS to examine work hardening loss. The weld length was 150 mm, produced using an H11 steel tool with a shoulder diameter of 11.48 mm, a pin diameter of 5 mm, and a pin length of 2.72 mm. The tool was operated without a tilt angle (0° tilt) throughout the process. A plunge depth of approximately 0.1 mm was applied to ensure sufficient shoulder contact during welding. After welding, the resulting plate measured 200 mm × 200 mm, with a thickness of 3.0 mm for the AA5052 and 3.2 mm for the AA6061. This thickness mismatch was intentionally retained to examine whether full penetration and sound joint formation could still be achieved under slightly asymmetric thickness conditions. A thin spacer was placed beneath the thinner plate to maintain top-surface alignment throughout the process. The full set of welding parameters is summarised in Table 2, and the welded sample is shown in Figure 1. Welding parameter sets 1–5 were designed to maintain approximately similar heat-input characteristics, expressed in terms of a near-constant k-factor (TTS/TRS), and to observe the effects of TTS and TRS on temperature. To evaluate the weld quality, three identical samples were extracted from the start, middle, and end regions of weld Set 3 for microstructural analysis. For hardness measurements, samples of the same size were taken from all five weld sets.

Two K-type thermocouples (temperature rating: −200 to 1100 °C [34]) were embedded along the weld line to record transient temperatures at different locations. Their positions were measured relative to the plate left edge, where the longitudinal direction corresponds to the welding direction and the transverse direction is perpendicular to it. The positions of the thermocouples are shown in Figure 1b and summarised in Table 3. To validate the temperature model, thermocouples were installed for five set groups. However, due to a malfunction, experimental data for thermocouple 2 were lacking in Set 5. Therefore, validation for Set 5 was conducted using thermocouple 1 only.

Before microstructure observation and the Vickers microhardness test, all samples underwent standardised surface treatment procedures to ensure the reliability of imaging and reduce artefacts. Silicon carbide (SiC) sandpaper up to 4000-grit was utilised for mechanical grinding to remove processing damage and surface irregularities. Subsequently, a 0.05 µm colloidal silica suspension was used for polishing to obtain a smooth and reflective surface, which was suitable for optical and electron microscopy observation. To use scanning electron microscopy (SEM) and scanning electron microscopy–electron backscatter diffraction (SEM-EBSD) for advanced characterisation, these samples were vibratory-polished for two hours in the same colloidal silica suspension for a mirror-like finish. After that, samples for EBSD analysis underwent ion beam polishing at 2 keV for two hours using the Gatan PIPS-II system (Gatan, Inc., Pleasanton, CA, USA). This final ion beam polishing step is crucial for the elimination of surface artefacts and oxide layers, which can improve the indexing quality of EBSD.

Optical microscopy used the Hirox MXB-2500REZ (Hirox Co., Ltd., Tokyo, Japan) to detect defects in the sample, such as voids, cracks and inclusions. Zeiss ULTRA Plus SEM (Carl Zeiss Microscopy GmbH, Oberkochen, Germany) equipped with a Symmetry CMOS EBSD detector (Oxford Instruments, High Wycombe, UK) was used for SEM and EBSD analysis of the selected area. The step size was set to 1.7 μm, and the acceleration voltage range was 20 to 30 kV. Data collection was performed using Oxford Instruments AZtec software (https://nano.oxinst.com/products/aztec/, accessed on 15 January 2026), and the minimum acquisition time for each data point was 0.3 ms. The EBSD results were used to generate inverse pole figure (IPF) maps, which were used to recognise the changes in texture and orientation of different welding zones. The local mechanical properties of the weld were evaluated by the Vickers microhardness test. Three samples for testing from the starting, middle and end sections of the weld of Set 3 were prepared. They were measured with Struers DuraScan-80 automatic Vickers hardness tester (Ballerup, Copenhagen, Denmark) in accordance with the ASTM E92-23 standard [35], with the load of 500 g (HV_0.5_) and a pressure dwell time of 10 s and the indentation spacing of 0.4 mm, which met the minimum requirement of at least four times the diagonal length of the indentation.

### 2.2. Model

**Figure 2:**
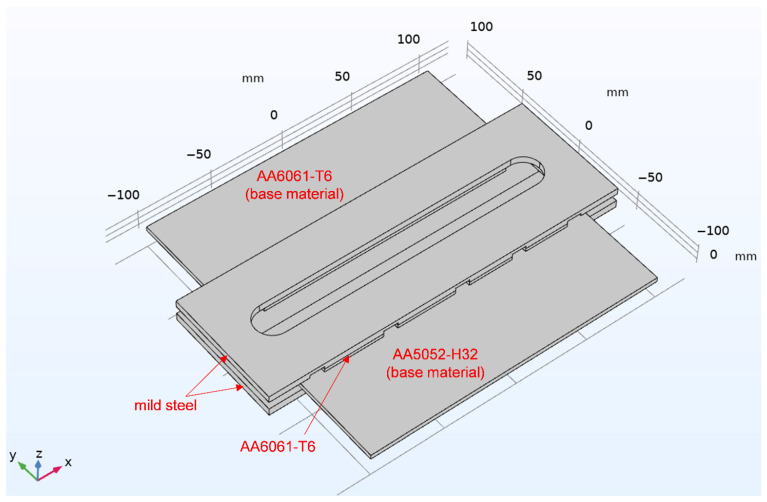
Numerical model geometries and structures of the FSW workpiece (all dimensions in mm).

Based on the experimental configuration of AA6061-T6 and AA5052-H32, a workpiece measuring 200 mm × 200 mm × 3.2 mm was modelled using COMSOL Multiphysics, as shown in Figure 2. A mild steel plate measuring 244 mm × 83 mm × 5.2 mm was placed beneath the workpiece to provide mechanical constraint and prevent rigid-body motion during welding. Positioned above were a 170.2 mm × 83 mm × 2.4 mm AA6061-T6 aluminium plate and a 244 mm × 83 mm × 4.6 mm steel plate, which together acted as a clamping system to stabilise the workpiece and maintain its position throughout the welding process. Five square openings on the side of the AA5052 were used to install thermocouples for temperature measurement.

According to [31], the fundamental partial differential equation governing heat transfer and thermal energy conservation during the FSW process within the workpiece plate is formulated as

(1) $$\rho C_{p}\frac{\partial T}{\partial t}=\nabla \cdot \left(k\nabla T\right)-\rho C_{p}u\cdot \nabla T+Q$$

where ρ is the density of the workpiece material, $C_{p}$ is the heat capacity, *T* is the temperature field, *t* is the time, *k* is the heat conductivity, and *u* is the welding speed of the tool.

During the FSW process, the heat generated from both frictional contact and plastic deformation rapidly diffuses through the aluminium plates. Simultaneously, heat dissipation occurs at the top and bottom surfaces primarily due to natural convection and radiation to the ambient environment. As presented by [36], the corresponding heat flux boundary conditions at these surfaces are expressed in Equations (2) and (3).

(2) $$q_{top}=h_{up}\left(T_{0}-T\right)+\varepsilon \sigma \left(T_{amb}^{4}-T^{4}\right)$$

(3) $$q_{bottom}=h_{down}\left(T_{0}-T\right)+\varepsilon \sigma \left(T_{amb}^{4}-T^{4}\right)$$

where $h_{up}$ and $h_{down}$ are heat transfer coefficients for natural convection from the upside and downside, respectively; $T_{0}$ is accompanying reference temperature; ε is surface emissivity; σ is the Stefan–Boltzmann constant, and $T_{amb}$ is ambient air temperature.

In this thermal model, the heat generated at the interface between the tool pin and the workpiece includes both surface friction and plastic deformation heat [37]. This interaction is described by the surface heat flux in Equation (4). To better represent the heat input from the rotating pin, a simplified thermal model was developed in COMSOL. Following the method in [38], a Gaussian volumetric heat source was applied to describe the heat generated by the pin stirring, as shown in Equation (5).

(4) $$q_{pin}\left(T\right)=\frac{\mu }{\sqrt{3\left(1+\mu^{2}\right)}}r_{p}\omega Y\left(T\right)$$

(5) $$Q_{pin}\left(x,y,z\right)=\frac{A_{pin}\times q_{pin}\left(T\right)}{\pi \delta r_{p}^{2}}exp\left(\frac{-2\left(x^{2}+y^{2}\right)}{r_{p}^{2}}\right)exp\left(\frac{-\left|z\right|}{\delta }\right)$$

where $q_{pin}$ is pin heat flux (W/m^2^), μ is friction coefficient between the pin and workpiece, $r_{p}$ is pin radius, ω is angular velocity (rad/s), $Y\left(T\right)$ is the average shear yield stress of the base materials as a function of temperature, $Q_{pin}$ is the volumetric heat flux (W/m^3^), $A_{pin}$ is the lateral surface area of the pin, δ is the pin length, and |*z*| is the absolute value of the z-coordinate.

The interface between the shoulder and the workpiece serves as a significant source of heat generation. The local surface heat flux per unit area (W/m^2^), which depends on the radial distance from the tool centre, is given by the equation proposed by Colegrove [37], as shown in Equation (6).

(6) $$q_{shoulder}\left(r,T\right)=\left\{\begin{array}{cc} \mu \left(\frac{F_{n}}{A_{shoulder}}\right)\omega r & ;\;if\;T<T_{melt}\\ 0 & ;\;if\;T>T_{melt} \end{array}\right.$$

where $F_{n}$ is normal force, $A_{shoulder}$ is surface area of shoulder, and $T_{melt}$ is aluminium melting temperature.

Table 4 summarises the main process parameters used in the thermal simulation. These parameters were selected based on experimental conditions and relevant literature to ensure consistency between the numerical model and actual welding conditions.

**Figure 3:**
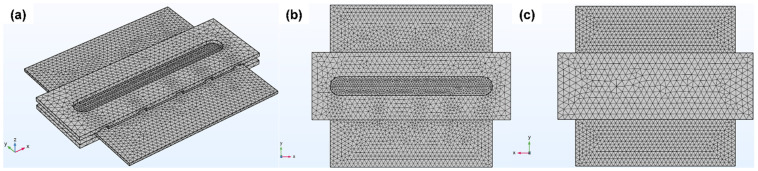
Finite element mesh of the FSW model: (**a**) isometric view; (**b**) top view; and (**c**) bottom view.

A three-dimensional free tetrahedral mesh was used for the thermal analysis, as shown in Figure 3. The mesh consisted of 28,173 elements and 7518 nodes, providing sufficient resolution for accurate field computation. It was refined along the weld path to accurately capture the steep temperature and temperature gradients, while a coarser mesh was applied in regions away from the heat-affected zone to reduce computational cost. The element size ranged from 0.56 mm to 2.94 mm in the weld region and up to 19.5 mm in the outer regions to ensure both accuracy and computational efficiency.

## 3. Results and Discussion

### 3.1. Simulated Thermal Field

**Figure 4:**
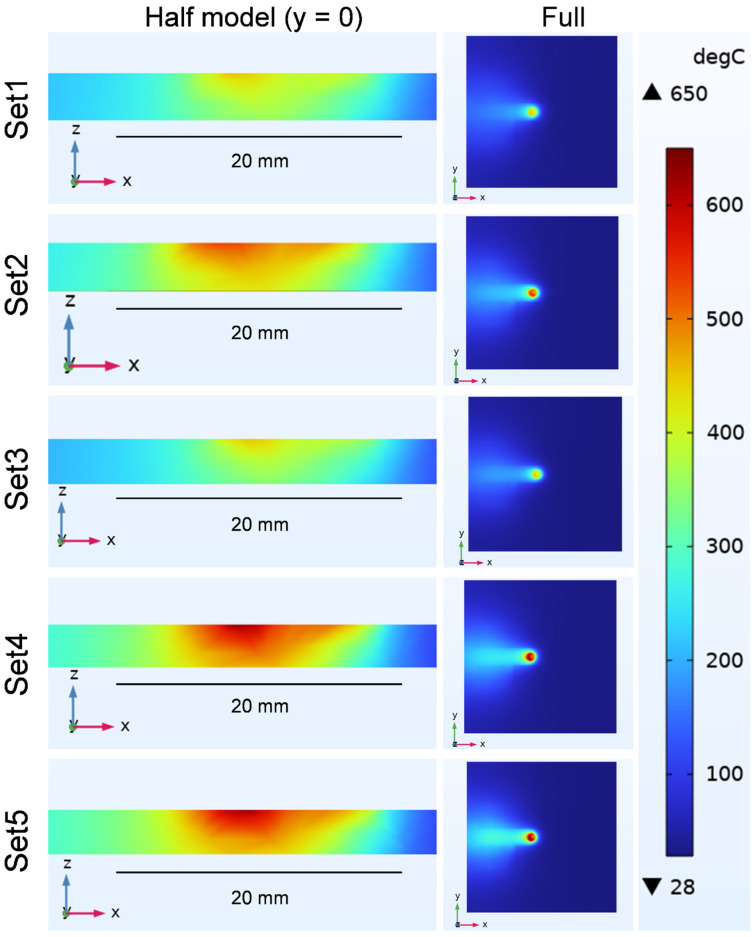
Simulated temperature fields at the mid-weld position during steady-state FSW under different welding parameter sets, shown using both half and full models.

Figure 4 shows the simulated temperature field when the tool reaches the mid-weld position under different welding parameter sets, including the half model and the full model. At this stage, the peak temperature represents the maximum temperature experienced by the entire welded plate under steady-state welding conditions. As expected, the maximum temperature is located on the plate surface near the shoulder–pin interface, forming steep thermal gradients within the stir zone due to concentrated heat input from friction and plastic deformation [39]. Among the five parameter sets, Set 4 exhibited the highest instantaneous peak temperature at 641 °C near the shoulder-pin interface, which was attributed to its higher TRS and the resulting increased heat input. In contrast, Set 3 showed the lowest instantaneous peak temperature at only 455 °C, corresponding to lower heat input due to a lower TRS. Although the instantaneous peak temperatures of all five sets are below the melting points of AA6061-T6 (652 °C) and AA5052-H32 (649 °C), Set 4 approaches the melting temperature. This difference in thermal history provides a direct explanation for the more pronounced softening observed in Set 4 compared with Set 3, while confirming the solid-state nature of the FSW process.

### 3.2. Validation of the Thermal Field with Experimental Results

**Figure 5:**
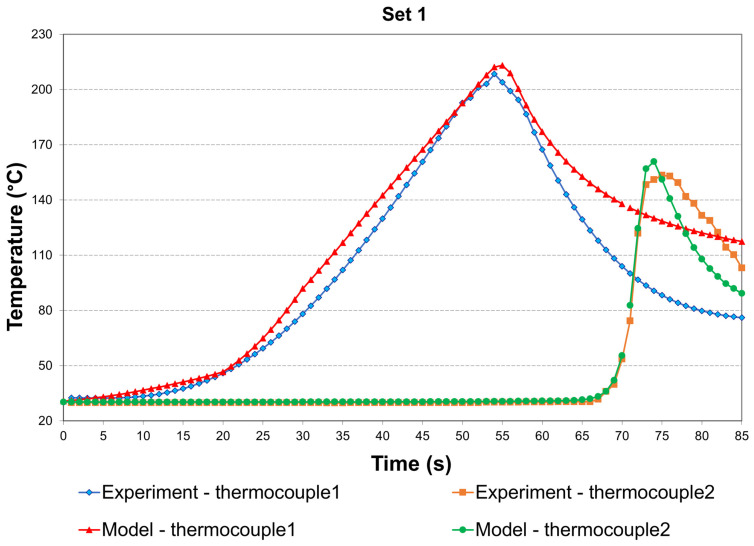
Comparison of experimental and simulated temperature histories at the two thermocouples of Set 1.

Figure 5 compares the simulated and experimental temperature histories measured by the two thermocouples in Set 1. A quantitative agreement is observed between the COMSOL predictions and the experimental measurements, with the simulated curves closely capturing the experimentally measured peak temperatures within a reasonable margin. The simulated temperature at thermocouple 2 matches the experimental result more closely, whereas thermocouple 1 shows a small deviation. This difference is mainly due to the complex contact conditions during preheating, which were simplified in the model. In addition, during the experiments, the thermocouples were wrapped and fixed using thermally conductive adhesive, thereby increasing the local heat capacity. The idealised convection boundary conditions adopted in the simulation also led to discrepancies between the simulated and experimental temperature responses, especially during the cooling phase.

Table 5 summarises the simulated and experimental peak temperatures of the two thermocouples for the remaining four parameter sets. For all cases, the errors between the simulation and experiment are within 10%. The temperature evolution is mainly influenced by the combined TTS and TRS, with their relative contributions depending on the parameter set. For Sets 2 and 5, the TRS values are the same, but Set 5 has a higher TTS, resulting in a shorter tool dwell time during welding. Therefore, a lower peak temperature is recorded at thermocouple 1 in Set 5. For Sets 3 and 4, the TTS is the same, but Set 4 has a higher TRS, which increases the plastic deformation of the material. Consequently, the peak temperatures measured by both thermocouples in Set 4 are higher than those in Set 3.

### 3.3. Correlation Between Thermal History and Microstructural Evolution

To establish the correlation between thermal history and microstructure, optical microscopy was performed on samples taken from the start, middle, and end of the weld in Set 3. These three locations were selected to capture the change in material flow and thermal exposure along the tool path, as the thermal and mechanical conditions vary significantly during tool entry, steady-state welding, and tool exit. Set 3 was selected for optical microscopy because it produced a sound, defect-free joint under a relatively stable thermal condition within the tested parameter matrix, making it ideal for linking temperature effects to microstructural outcomes.

**Figure 6:**
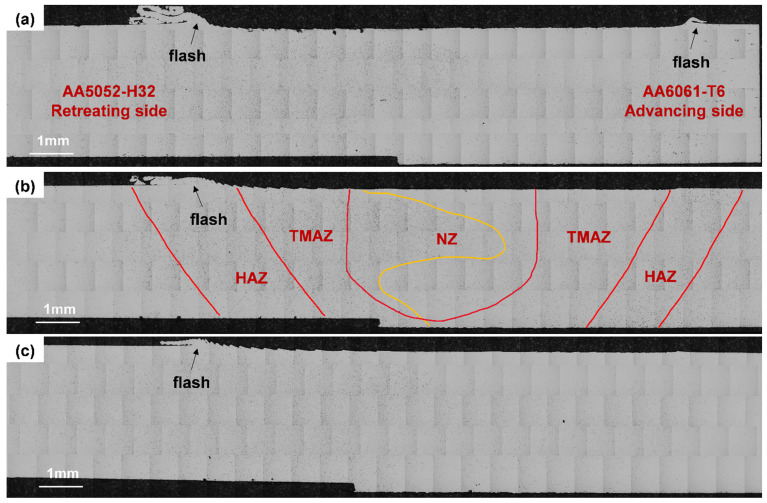
Optical micrographs of the FSW joint at three locations of Set 3: (**a**) start; (**b**) middle; and (**c**) end of the weld.

Figure 6 presents optical microscopy images of FSW joints at the start, middle, and end of the weld of Set 3. No porosity or cracking was observed in either aluminium alloy at the three locations. The NZ, TMAZ, and HAZ in Figure 6b were initially delineated based on the observed plastic flow morphology and their relative spatial positions across the weld cross-section. This visual identification was subsequently verified by quantitative analyses, including simulated temperature distributions, EBSD characterisation, and local hardness measurements presented in later sections, thereby reducing subjectivity in zone classification. Flash formation was observed on both sides of the weld, especially on the AA5052 side. This behaviour is attributed to the difference in the mechanical properties of the two alloys, because AA5052-H32 exhibits a lower flow stress, allowing plastically softened material to be more readily expelled from the weld region. In addition, excessive softening caused by locally high heat input or increased tool plunge depth during the initial dwell period and transient tool entry stage may further promote flash formation. During welding, plastically softened material was squeezed out from beneath the tool shoulder and accumulated along the weld edges, forming surface flash [40]. The use of a flat shoulder makes this more pronounced, as it does not effectively restrict the flowing material [41]. At the weld start position (Figure 6a), flash formation was most pronounced on both the AS and RS. However, its extent gradually decreased as the tool traversed along the joint and steady-state welding conditions were established.

**Figure 7:**
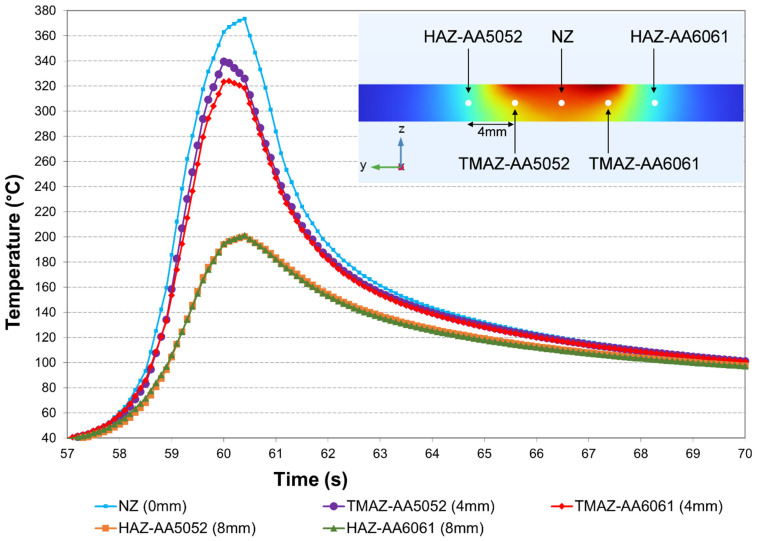
Simulated temperature–time histories at five representative locations at the mid-thickness plane of the weld cross-section at the weld centre for Set 3.

Figure 7 shows the simulated thermal histories at five representative locations in Set 3. These points were selected at the mid-thickness plane of the weld cross-section at the weld centre, rather than on the plate surface. They were chosen to capture the typical thermal gradients across the weld: the NZ point corresponded to the exact weld centre; the two TMAZ points were located 4 mm from the weld centre on the AA5052 and AA6061 sides, respectively; and the two HAZ points were positioned 8 mm from the centre on both sides. The results show that the NZ experienced the highest peak temperature at 374 °C. As the distance from the centre of the weld increased, the temperature of the TMAZ and HAZ gradually decreased. Within the TMAZ, the peak temperature on the AA5052 side was 16 °C higher than that on the AA6061 side. This difference is attributed to the lower thermal conductivity of AA5052, which retains more heat. In contrast, the HAZ on both sides experienced relatively weaker thermal influence, resulting in nearly overlapping temperature curves. It should be noted that, because all five locations are positioned at the mid-thickness plane, their peak temperatures are lower than the maximum temperature of the welded plate in Figure 4, which occurs at the top surface near the shoulder-pin interface. Nevertheless, the relative differences in thermal histories captured at these mid-thickness locations provide a representative measure of local thermal exposure across the weld region, which directly contributes to the distinct grain structures later observed in the corresponding microstructural regions.

**Figure 8:**
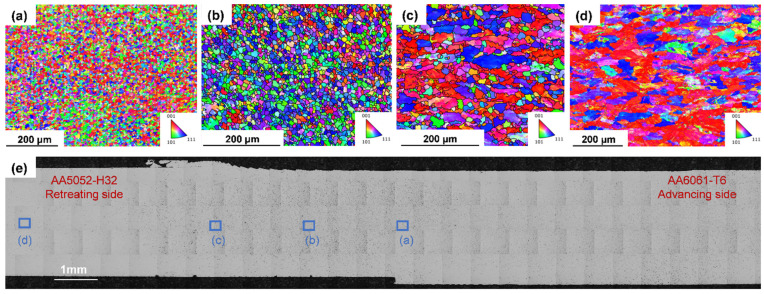
EBSD images of AA5052-H32 at the middle position of the weld in Set 3: (**a**) near NZ, (**b**) near TMAZ, (**c**) near HAZ, and (**d**) BM. The cworresponding image locations are indicated in (**e**).

Figure 8 presents EBSD IPF-Z maps of the AA5052-H32 at the middle position of the weld in Set 3. In the IPF-Z configuration, the *Z*-axis was normal to the observed surface, and the colour coding indicated crystallographic orientations: blue corresponds to the ⟨111⟩ direction, red to ⟨001⟩, and green to ⟨101⟩. This mapping facilitated the visualisation of texture development and microstructural refinement induced by the FSW process. Figure 8a shows EBSD images of near NZ at the weld middle. This region exhibited a random distribution of crystal orientation, and its grain morphology differed significantly from that of the parent material (Figure 8d). These differences arose from the intense plastic deformation and frictional heat generated during welding, which disrupted the original grain structure and promoted DRX. As a result, fine and equiaxed grains were formed throughout the NZ [42,43]. The area-weighted average grain diameter at this area was 11.09 µm, and the smallest grain was only 6.07 µm, suggesting that the average grain diameter was even smaller in the NZ region. The plastic deformation increases the dislocation density, thereby improving DRX and contributing to the formation of finer equiaxed grains [44]. Figure 8b depicts EBSD maps near the TMAZ at the middle of the weld, located on the AA5052-H32 side. It exhibited grain refinement, with an area-weighted average diameter of 12.76 µm. This value was significantly lower than that in the parent material, indicating that although this region underwent plastic deformation during FSW, the strain and temperature were generally insufficient to trigger full DRX [8]. Therefore, the original grains were deformed and rotated, but they were not entirely replaced by new recrystallised grains. The previously rolled grains were transformed into morphologies closer to equiaxed. In addition, the crystal orientation has also changed, and more grains were oriented along the ⟨111⟩ axis. It should be noted that this observation is based solely on IPF-Z mapping and reflects texture evolution rather than a direct assessment of slip behaviour.

In contrast, the area-weighted average grain diameter near the HAZ in Figure 8c was 28.31 µm, which was higher than the grain diameters near the TMAZ and near the NZ, reflecting the gradual increase in the grain size towards the outer edge of the weld zone. This increase was mainly driven by heat, because this area was mainly affected by the thermal cycle of welding, and there was no direct tool effect; thus, mechanical deformation could be ignored. Therefore, the microstructure of this area was similar to that of the BM. The crystal orientation of these regions was mainly arranged in the ⟨001⟩ and ⟨111⟩ directions. Compared to other regions, the grain orientations in HAZ are similar [45,46], and the grain texture exhibits strong anisotropy. This observation confirmed that the friction stirring effect weakened rapidly outside the TMAZ-HAZ interface and that the grain structure was mainly affected by thermal exposure and plastic deformation of the tool pin and shoulder. As shown in Figure 8d, the AA5052-H32 alloy exhibits a mixed orientation dominated by red and blue colours, corresponding to grains primarily oriented along the ⟨001⟩ and ⟨111⟩ directions. The area-weighted average equivalent circular diameter (ECD) is 48.37 µm.

### 3.4. Correlation Between Thermal History and Microhardness

**Figure 9:**
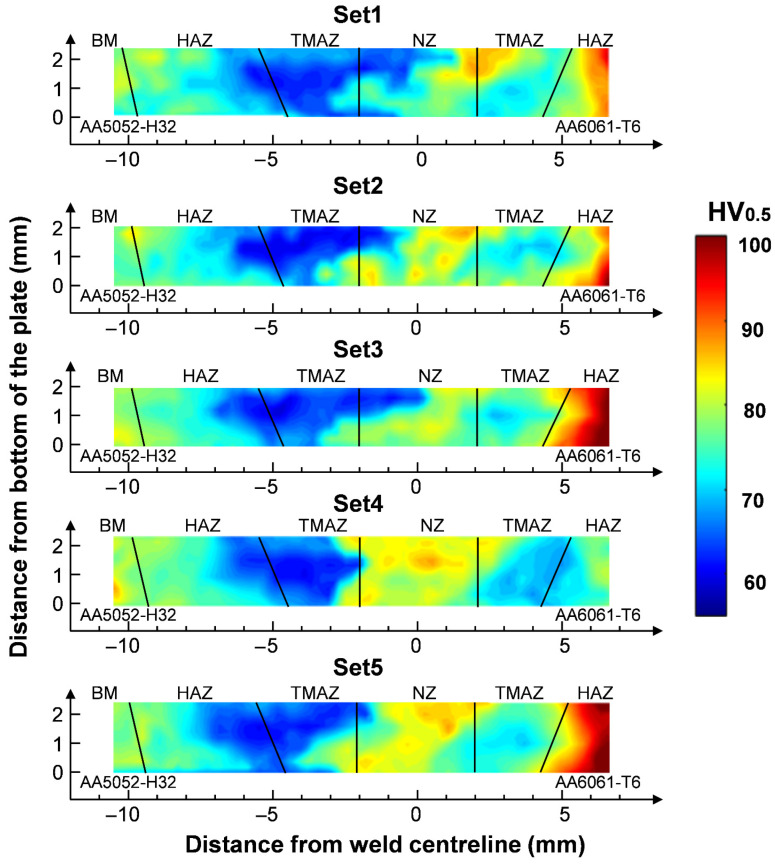
Vickers hardness (HV_0.5_) distribution maps under different welding parameter sets.

Figure 9 illustrates the hardness distributions for the five welding parameter sets. Across all conditions, a distinct low-hardness band consistently formed in the TMAZ on the AA5052-H32 side, indicating that thermal softening of the cold-worked AA5052-H32 governed the weakest region of the dissimilar joint, which is mainly due to recovery and associated work-hardening losses. Among the tested parameters, Set 3 exhibited the lowest hardness value in the TMAZ (58.2 HV_0.5_).

Within the weld zone, Set 4 exhibited the highest average hardness in the NZ, approximately 85 HV_0.5_. This is consistent with the higher peak temperature predicted for Set 4 (641 °C), which, together with intense plastic deformation, promoted DRX and produced fine equiaxed grains. According to the Hall–Petch relationship, grain refinement increased the hardness by increasing the grain boundary area, thus hindering dislocation movement [47,48]. Asymmetric flow could also lead to differences in the hardness of the advancing and retreating sides, because differences in heat generation and plastic deformation changed the degree of recrystallisation and precipitation phase transformation. The blue regions in the hardness maps represented softened zones predominantly located in the TMAZ/HAZ and, in some cases (e.g., Set 1 and Set 3), extending slightly into the NZ. Since the joint strength was governed by the weakest region, these softened zones were expected to determine the overall mechanical performance, with tensile failure likely initiating in these regions.

**Figure 10:**
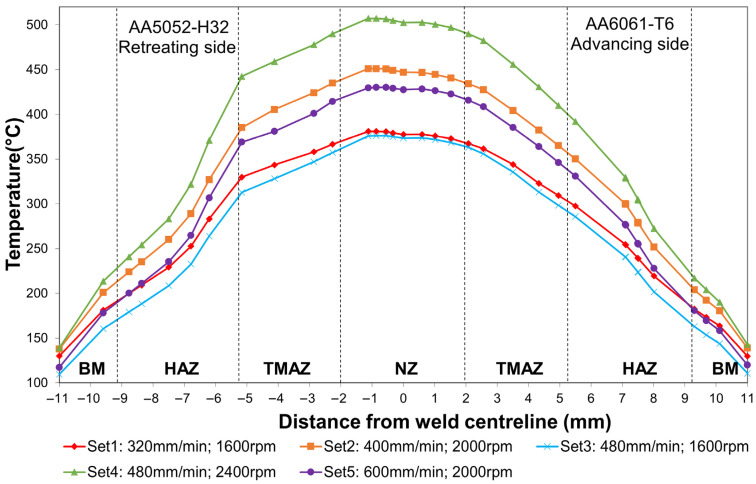
Temperature distribution along the centreline of the weld cross-section at the mid-weld position (75 mm from the weld start) under different welding parameter sets.

To establish the relationship between thermal history and hardness evolution, the temperature variation across the weld cross-section was first examined, as shown in Figure 10. The temperature profiles were extracted along the centreline of the weld cross-section when the tool reached the mid-weld position, corresponding to a distance of 75 mm from the weld start. At this same mid-weld and mid-thickness location, Vickers microhardness measurements were subsequently performed in the following section to enable a direct correlation with the local thermal history. The highest temperatures for both alloys were located at the NZ/TMAZ interface, at the edge of the tool pin, where the material directly underneath experienced the greatest frictional heat generation and plastic deformation. The AA5052 side consistently reached higher temperatures due to its lower thermal conductivity, resulting in more severe softening on the RS. Set 4 produces the highest peak temperature (507 °C), whereas Set 3 shows the lowest (376 °C). At a constant traverse speed, increasing the rotational speed enhances frictional work and plastic deformation, leading to higher heat input [49]. When rotational speed is constant, as in Set 1 and Set 3, the higher traverse speed in Set 3 reduces dwell time and results in lower temperatures [25]. The influence of rotational speed on heat input is greater than that of traverse speed, which is consistent with the findings reported by El-Zathry et al. [7].

**Figure 11:**
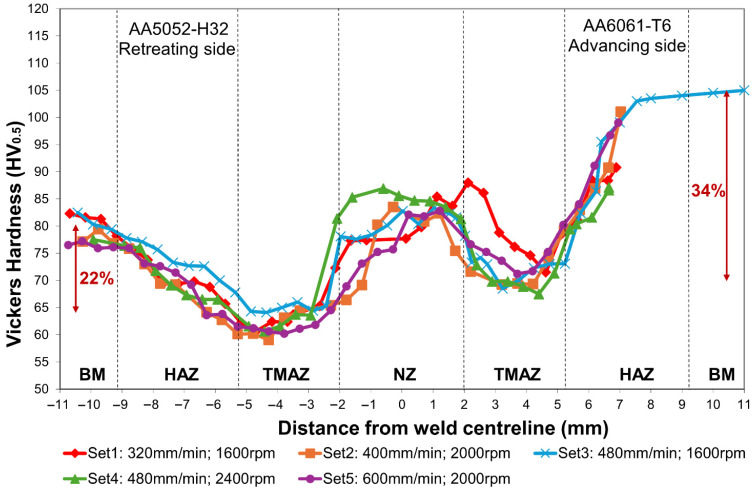
Vickers microhardness profile along weld centreline under different welding parameter sets.

Figure 11 shows the Vickers hardness distribution along the centreline of the parameter groups. On the AA5052-H32 side, the lowest hardness was found in the TMAZ at −4 to −3 mm, with values between 59 and 64 HV_0.5_. AA5052-H32 is a non-heat-treatable alloy and derives its strength primarily from solid solution strengthening and work hardening. In the TMAZ, elevated temperatures near those of the NZ led to loss of cold work strengthening. However, the temperature and plastic deformation were insufficient to fully induce recrystallisation; as a result, the Hall–Petch strengthening due to limited grain refinement was inadequate to compensate for the reduction in other strengthening mechanisms. Meanwhile, high temperature reduced dislocation density, further decreasing hardness. In contrast, the HAZ, which experienced thermal exposure without mechanical deformation, retained some of the cold-worked microstructure and strengthening phases, resulting in higher hardness than the TMAZ.

On the AA6061-T6 side, the TMAZ at +3 to +4 mm also exhibited the minimum hardness, ranging from 68 to 71 HV_0.5_. The precipitation sequence in Al-Mg-Si 6xxx alloys progresses from a supersaturated solid solution to Guinier–Preston zones, then to metastable β″ and β′ phases, and finally to the stable β (Mg_2_Si) phase [50]. As a heat-treatable alloy, AA6061-T6 is strengthened primarily through precipitation hardening, particularly through fine β″ precipitates. During FSW, thermal cycling causes needle-shaped β″ to dissolve or coarsen, transforming them into rod-shaped β′ precipitates, significantly reducing the alloy strengthening capability [51]. In the HAZ, grain coarsening and over-ageing further contribute to softening [52]. As grain size increases, hardness decreases due to the inverse relationship described by the Hall–Petch effect.

Quantitatively, the hardness of AA5052-H32 base material is 80 HV_0.5_, with an approximately 22% drop observed in the TMAZ. For AA6061-T6, the base material hardness is 105 HV_0.5_, with a more substantial about 34% drop in the TMAZ. This larger reduction was attributed to the thermal sensitivity of the precipitation-hardening mechanism in AA6061-T6, whereas the softening in AA5052-H32 was primarily due to recovery of dislocation structures, with the fundamental strengthening mechanism largely retained.

## 4. Conclusions

### 4.1. Findings

This research successfully welded AA5052-H32 and AA6061-T6 joints using FSW joints and developed a thermal simulation model of the FSW process using COMSOL Multiphysics. The main research conclusions are summarised below:

1. The COMSOL thermal model was validated against experimental thermocouple measurements. For Set 1, the simulated and experimental temperature curves almost overlapped, while for Sets 2–5, the predicted peak temperatures agreed with the experimental values within an error of 10%.
2. The simulated peak temperatures of the entire welded plate varied from 455 °C to 641 °C across different welding parameter sets. All values remained below the melting temperatures of both AA5052-H32 and AA6061-T6.
3. At the analysed locations (start, middle, and end), the welding conditions of 480 mm/min, 1600 rpm, and a plunge force of 4204 N resulted in effective material mixing with no observable defects. Thermal history–microstructural evolution correlations were identified across the NZ, TMAZ, and HAZ at the middle weld position. DRX occurred in the NZ, forming fine equiaxed grains with a minimum grain diameter of 6.07 µm, approximately eightfold smaller than that of the BM AA5052-H32. Partial recrystallisation was observed in the TMAZ, while the HAZ exhibited larger anisotropic grains dominated by thermal exposure.
4. A thermal history–hardness distribution relationship was identified. The minimum hardness occurred in the TMAZ on the AA5052-H32 side, showing a 22% reduction compared with AA5052 due to recovery and grain growth, while the TMAZ hardness on the AA6061-T6 side decreased by 34%. The work-hardening response of AA5052-H32 and the precipitation strengthening of AA6061-T6 led to different degrees of softening under thermal exposure.

### 4.2. Recommendation for Future Work

Future work should include comprehensive mechanical testing to establish the relationship between microstructure and joint strength in dissimilar FSW joints. The numerical model can be extended to incorporate elastic–plastic behaviour, residual stress, and welding-induced distortion under realistic boundary conditions, with experimental validation. In addition, integrating machine learning with experimental and numerical data may enable predictive modelling and adaptive process control for broader engineering applications of FSW.

## Figures and Tables

**Table 1:** Chemical composition of AA6061-T6 and AA5052-H32.

Alloys	Mg	Mn	Cu	Cr	Si	Fe	Zn	Ti	Al
AA6061-T6	1.20	0.15	0.20	0.40	0.60	0.75	-	-	Balance
AA5052-H32	2.48	0.071	0.092	0.10	0.092	0.388	0.008	0.03	Balance

**Table 2:** The welding parameters for FSW process.

Set	TTS (mm/min)	TRS (rpm)	Plunge Force (N)
1	320	1600	3600
2	400	2000	3799
3	480	1600	4204
4	480	2400	3791
5	600	2000	4180

**Table 3:** Thermocouple positions measured from the weld centre using ImageJ (https://imagej.net/ij/download.html, accessed on 15 January 2026).

Position	Thermocouple 1	Thermocouple 2
Longitudinal direction (mm)	15	155
Transverse direction (mm)	−11	−11

**Table 4:** The summarised welding input parameters used in this model.

Name	Description	Value	Unit
*T*_0_	Ambient temperature	301.15	K
*T_melt_*	Melting temperature of the workpiece	933	K
*h_up_*	Heat transfer coefficient on the upside of the workpiece	20	W/(m^2^·K)
*h_down_*	Heat transfer coefficient on the downside of the workpiece	10	W/(m^2^·K)
ε	Surface emissivity	0.3	-
μ	Coefficient of friction	0.4	-
*r_s_*	Shoulder radius	5.74	mm
*r_p_*	Pin radius	2.0	mm
δ	Pin length	2.72	mm
*A_shoulder_*	Surface area of the shoulder	90.9	mm^2^

**Table 5:** Experimental and simulated peak temperatures measured by thermocouples.

Set	Thermocouple 1			Thermocouple 2		
	Experiment (°C)	Model (°C)	Error (%)	Experiment (°C)	Model (°C)	Error (%)
2	210	220	4.8	154	160	3.9
3	168	183	8.9	109	115	5.5
4	232	248	6.9	152	158	3.9
5	177	193	9.0	-	-	-

## Data Availability

The original contributions presented in this study are included in the article. Further inquiries can be directed to the corresponding author.
